# Insights from Subsurface Monitoring for Engineering of the Stimulation Pattern in Fractured Reservoirs

**DOI:** 10.1007/s00603-025-04525-5

**Published:** 2025-05-25

**Authors:** Nima Gholizadeh Doonechaly, Kai Bröker, Marian Hertrich, Martina Rosskopf, Anne Obermann, Virginie Durand, Francisco Serbeto, Alexis Shakas, Xiaodong Ma, Antonio Pio Rinaldi, Victor Clasen Repollés, Linus Villiger, Men-Andrin Meier, Valentin Gischig, Katrin Plenkers, Hansruedi Maurer, Stefan Wiemer, Domenico Giardini

**Affiliations:** 1 Center for Hydrogeology and Geothermics (CHYN), University of Neuchâtel, Neuchâtel, Switzerland; 2 Department of Earth and Planetary Sciences, ETH Zurich, Zurich, Switzerland; 3 Swiss Seismological Service (SED), ETH Zurich, Zurich, Switzerland; 4 Côte d’Azur Université, Nice, France; 5 Geo-Energie Suisse AG, Zurich, Switzerland; 6 School of Earth and Space Sciences, University of Science and Technology of China, Hefei, China; 7 GmuG, Bad Nauheim, Germany

**Keywords:** EGS, Hydraulic stimulation, Induced seismicity, Kaiser effect, Pressure compartments, Slip tendency, Fractured reservoirs

## Abstract

Stimulation operations enhance the performance of geothermal reservoirs by enhancing fluid flow and heat transfer. Predicting stimulation outcomes is challenging due to the complexity of reservoir properties and limited observations given by operational conditions. The stress state, natural geological structures, pressure distribution, and injection protocols play crucial roles in the engineering of a stimulation operation. This study provides in-depth observations from a hectometer-scale stimulation experiment conducted at the Bedretto Underground Laboratory for Geosciences and Geoenergies within a densely monitored crystalline rock volume with an overburden of more than 1 km. We found that hydraulic connectivity, pressure compartments, and the geomechanical characteristics of existing geological structures play important roles in the propagation patterns of seismic events. Notably, the initiation and distribution of seismicity are markedly influenced by the zonal pressure isolation and the propagation of nominal pressure diffusion fronts across the reservoir. Our findings highlight the necessity of adapting stimulation strategies according to the unique geomechanical and geological characteristics of the reservoir. This claim is supported by the distinct activation patterns observed between the first and second injection cycles in the current case study. The spatial extent of the stimulated volume can be partially controlled by the number of stimulation cycles and injection pressure level, as farther structures are more likely to be activated in the subsequent cycles. The results also indicate that the Kaiser effect can be attenuated due to changes in the flow pathway and stress caused during stimulation, consistent with a proposition from a recent study. Our findings underscore the critical importance of the interplay between hydraulic pressures and stress states to optimize the stimulation of fractured reservoirs.

## Highlights

- Reservoir pressure compartments and their control via zonal isolation significantly influence seismic activity.
- Segments of seismic activity demonstrate a distinct backward propagation (from a distant location) towards the injection well, most likely influenced by a compartmentalized pressure system.
- The Kaiser effect influences seismic activity across two injection cycles.

## Introduction

Hydraulic stimulation, amongst all stimulation techniques, is the most commonly performed operation to improve the permeability of the subsurface reservoirs, in particular for developing Enhanced Geothermal Systems (EGS), by expanding the available surface area for flow and heat exchange (Jia et al. 2022; Lu 2018; Olasolo et al. 2016). Despite the long history of hydraulic stimulation, in particular hydraulic fracturing, since late 1940s (Testa 2019), predicting, characterizing, and engineering the stimulation pattern within the associated subsurface fracture network remains challenging due to the intrinsic complexity of the structures and limited available direct observations (Liang et al. 2023; Pollack et al. 2020; AbuAisha et al. 2016). A pore pressure increase within the reservoir volume initiates the reservoir stimulation process, which is often accompanied by induced seismic activity (Leonhardt et al. 2021; Schopper et al. 2020; Grigoli et al. 2018; Hillers et al. 2015; Obermann et al. 2015). In contrast to the common assumption of induced slip on pre-existing fractures playing a key role in the EGS stimulation operations, McClure and Horne (2014), along with subsequent studies by Villiger et al. (2020) and Villiger et al. (2021), suggested that mixed-mechanism stimulation (MMS), which involves both new and preexisting fractures, might be more plausible than previously assumed.

Stress regime analysis emphasizes fractures activated by the influence of the dominant reservoir stress field. While providing valuable information for designing and characterizing an EGS, induced seismicity presents potential hazards. Therefore, comprehensive monitoring and understanding of the associated seismo-hydromechanical processes are crucial not only for predicting the outcomes of hydraulic stimulation experiments (Schopper et al. 2020; Amann et al. 2018; Hillers et al. 2015) but also for mitigating induced seismicity, which has caused significant challenges in EGS projects, as evidenced by the events in Pohang, South Korea (2017), with a magnitude of 5.5, and in Basel, Switzerland (2006), where a series of induced events reached a magnitude of 3.4 (Ge and Saar 2022; Grigoli et al. 2018; Kim et al. 2018; Häring et al. 2008). Seismicity provides crucial insights into enhanced permeability within stimulated zones, although its use as a direct proxy might be overly simplistic due to underlying complexities in pore pressure dynamics and mechanical responses of the faults. Recent findings show the importance of variable propagation speeds of aseismic slip relative to the fluid front, which can be significantly influenced by pre-injection stresses and fluid injection parameters (Bhattacharya and Viesca 2019). Guglielmi et al. (2015) observed large permeability changes as a result of dilatant and slow aseismic slip, which was transitioned into a faster slip associated with micro-earthquakes. Moreover, Cappa et al. (2022) demonstrated that fault slip and opening can occur independently. Jacquey and Viesca (2023) further detailed the role of aseismic slip even in the post-injection phase, indicating that slip can still evolve after the pressurization stops. These studies may suggest that while microseismic activity can indicate regions of mechanical destabilization, it does not necessarily coincide with regions of enhanced permeability. Building on this understanding, the focus of recent research shifted toward understanding the flow path (Krietsch et al. 2020; Mukuhira et al. 2017a). Mukuhira et al. (2017b) utilized microseismic and stress data to estimate the increase in pore pressure needed during stimulation to induce shear slip along existing fractures. Their analysis showed that lower pressure propagates "farther and faster", while higher pore pressure propagates slower.

Efficient stimulation operations require not only a detailed understanding of reservoir properties and dynamics but also a detailed design of injection protocols based on specific reservoir properties (Lecampion 2021). Operational conditions can significantly affect the outcome of stimulation. As an example, several types of cyclic injection protocols have been utilized to increase the efficiency of stimulation operations (Li et al. 2021). Another important factor that influences the efficiency of stimulation operations is zonal isolation, which can be achieved for example, by implementing packers for the hydraulic separation of different flowing zones along the borehole (Meier et al. 2015). Ineffective completion and isolation between producing zones may lead to uncertainties in reservoir evaluation and unwanted cross-flow between different flowing zones, which can then lead to ineffective stimulation (Nozaki et al. 2019; Smith 1989).

Recent progress in reservoir surveillance techniques to monitor thermo-hydro-mechanical changes during induced seismicity, helps us to have a better understanding of the processes involved in hydraulic stimulation (Obermann et al. 2024; Houhou and Laloui 2022; Gischig et al. 2020). This paper explores the dynamics of reservoir stimulation through an in-depth analysis of seismic activity, pressure changes, and the in-situ stress regime in response to fluid injections. In this study, by evaluating the interactions between hydraulic connectivity and seismic propagation patterns during different injection cycles, we aim to provide suggestions on how to improve stimulation strategies for EGS. The study also emphasizes the role of the hydrostatic pressure system, the number of injection cycles, and the spatial relationship of activated structures to the injection interval. Our findings highlight the influence of pressure control and existing geological features on the stimulation patterns.

## Site Characterization

**Fig. 1:**
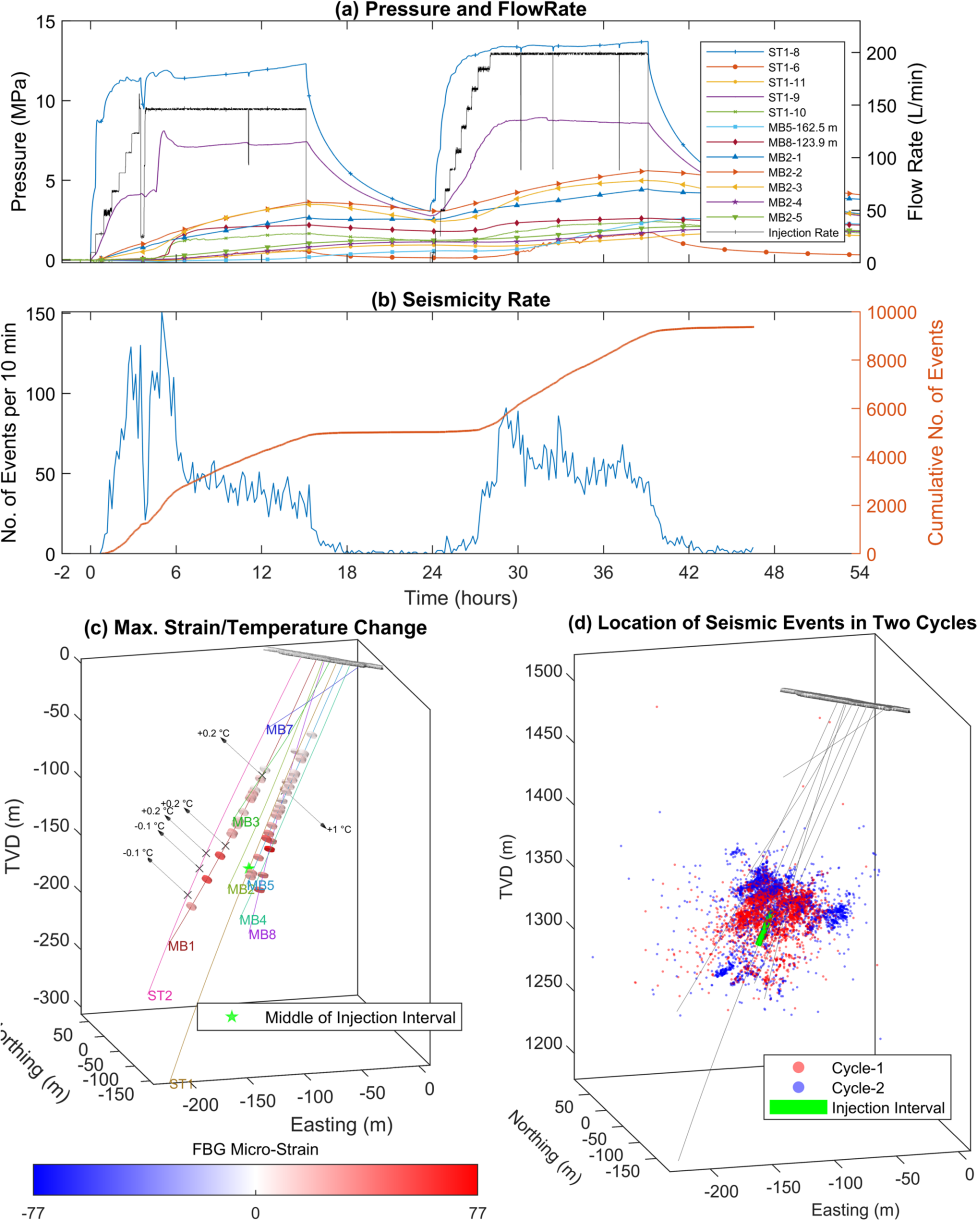
**a** Major pressure changes in the reservoir volume during the experiment, **b** the recorded seismicity rate and cumulative number of seismic events during the experiment, **c** a schematic representation of the BedrettoLab with the drilled boreholes along with the maximum FBG-strain/DTS-temperature changes (colorbar for strain is intentionally made symmetric so that white color represents no strain changes), and **d** the location of the detected seismic events colored based on their corresponding injection cycles (including the following shut-in periods). The zero-time in (**a**, **b**) represents the start of the first injection. The enlarged views of **c** including the packer depths in ST1 and MB2, are shown in Appendix 2. (Colour figure online)

The BedrettoLab, located at tunnel meter (TM) 2000 in the Bedretto tunnel in Switzerland, was established by ETH Zurich as part of Bedretto Underground Laboratory for Geosciences and Geoenergies (BULGG) for research in renewable geothermal energy as part of the Swiss Energy Strategy 2050 (SFOE 2021; Prognos AG 2012, BULGG). Bedretto tunnel consists of three geological units: Tremola series, Prato series, and Rotondo granite, the details of which are available in Rast et al. (2022), Wenning et al. (2022), and Keller and Schneider (1982). Nine boreholes (see Fig. 1c), ranging in measured depth from 101 to 404 m, have been drilled in the BedrettoLab for monitoring, characterization, and stimulation purposes, with a comprehensive characterization provided by Ma et al. (2022). The boreholes are equipped with various sensors, including pore pressure sensors, Acoustic Emission sensors (AE), piezoelectric accelerometers (ACC), fiber-optic cables for Distributed Temperature Sensing (DTS), Distributed Acoustic Sensing (DAS), Distributed Strain Sensing (DSS), and Fiber Bragg Grating (FBG), geophones, and ultrasonic transmitters (Plenkers et al. 2023). The spatial distribution of sensors and boreholes within the reservoir is depicted in Fig. 12 in Appendix 1. The sensor network design addresses installation challenges such as long and fractured boreholes, high pressure, and cementing requirements (Gholizadeh Doonechaly et al. 2024; Plenkers et al. 2023). ST1 is the main stimulation borehole and has an approximate average dip angle of 49.2°. It is zonally isolated with 14 packers for targeted stimulation operations (Meier et al. 2022). MB2 is the pressure monitoring borehole with six operational packed intervals, specifically designated for pressure monitoring purposes. Additionally, there are four grouted pressure sensors; two are located in MB5 and two in MB8 (Plenkers et al. 2023). All boreholes are characterized using geophysical logging and the details about the borehole-intersecting fractures in the reservoir volume can be found in Ma et al. (2022) and Bröker et al. (2024a).

### Injection Interval

This investigation focuses on the second phase of stimulation of interval 8 in ST1 due to several factors: exceptionally good sensor coverage for monitoring seismic activity, relatively high initial transmissivity, a large improvement in interval transmissivity resulting from stimulation operations relative to other intervals, and detected seismic events that distribute over a hectometer scale (Obermann et al. 2024). Interval 8 spans from a measured depth of 186.68–216.76 m from the top of the wellhead, corresponding to the true vertical depths (TVDs) of 135–157.7 m, respectively. For the sake of simplicity, all reported depths in this study refer to the measured depth unless otherwise stated. Interval 8 contains two distinct fracture zones extending from 205 to 209.5 m depth, with a prominent strike of about 230° and a dip between 43° and 70°. These fracture zones exhibit inflow, as observed in the spinner log, although no corresponding anomalies in temperature or conductivity were detected (Bröker et al. 2024a). In addition, numerous filled and undifferentiated fractures are present in this interval. Another notable inflow zone is evident at approximately 189 m, accompanied by a sharp increase in conductivity and a modest increase in the spinner log. At this depth, only a limited number of undifferentiated fractures were discovered. The center of interval 8 has an in-situ temperature of about 21.7 °C in static conditions. The details of the fracture system, including the above findings can be found in Bröker et al. (2024a).

### In-Situ Stress State

Bröker and Ma (2022) and Bröker et al. (2024b) estimated the stress magnitudes and orientations from mini-frac tests in and close to the BedrettoLab and their findings are as follows: *S*1, the maximum principal stress component, is aligned with the vertical overburden stress (ca. 26.5 MPa), although a slight inclination of a maximum of 10° is likely. The magnitude of the intermediate principal stress, *S*2 or SHmax, is close to the *S*1 magnitude (20.4–27.9 MPa) and trends between N100°–120°E (mean = N112°E). The minimum principal stress, *S*3 or Shmin, trends between N10°–30°E with a magnitude of 11.2–16.4 MPa. Also, they found that the stress state is on the transition between normal and strike-slip faulting (Shmin < SHmax ≤ Sv). Bröker et al. (2024b) have observed an anticlockwise rotation of the stress field by a maximum of 37° in a group of three shallow stress measurement boreholes (ca. 30 m deep) located within the BedrettoLab niche. This indicates a potentially heterogeneous stress field, which is locally influenced by fractures or fault zones (Bröker et al. 2024b). The influence of fault zones on the stress state was also investigated by Zhang et al. (2023), who analyzed borehole breakouts in the different monitoring and stimulation boreholes. They inferred a clockwise rotation of the stress field around the primary fault zone, accompanied by a decreasing stress ratio. It is plausible that the stress ratio, defined as *ϕ* = (SHmax − Shmin)/(SV − Shmin), decreases from its far-field value (ca. 0.9) to low values towards 0, due to the slip-induced stress drop near local fractures or fault zones (e.g., Shamir and Zoback 1992; Barton and Zoback 1994).

## Methodology

This study aims to analyze the stimulation patterns within interval 8 of ST1, by focusing on the complex interactions between seismicity and reservoir geomechanics. Utilizing seismic data processed by Obermann et al. (2024), coupled with the stress regime analysis provided by Bröker and Ma (2022) and Bröker et al. (2024b), and based on the impact of the preceding stimulation experiments as detailed by Gholizadeh Doonechaly et al. (2023) and Bröker et al. (2024a), we evaluate the detailed dynamic responses of the reservoir during stimulation.

### Experimental Approach

In the first stimulation phase (phase 1), referred to as the characterization phase, the upper eight intervals of ST1—that lay within a dense monitoring network—were stimulated with relatively low injection volumes to characterize the seismological and hydromechanical response of individual intervals (Bröker et al. 2024a; Obermann et al. 2024). These stimulations took place between November 2021 and March 2022. During phase 1, in interval 8, 4.8 m^3^ of water was injected during two injection cycles under pressure-controlled steps with a total injection time of ca. 3 h. The largest recorded injection flow rate was 100 L/min, and the maximum injection pressure reached 16.29 MPa. The hydromechanical analysis of the pressure and flow rate data showed a reactivation of the pre-existing structures likely occurring at 13.4 MPa (Bröker et al. 2024a). During this stimulation, 1289 seismic events were reliably detected and located (Obermann et al. 2024). The seismic activity propagated over 55 m, mainly around the upper part of the stimulated interval. It is interpreted as a fracture activated by stimulation. During the first injection cycle, the seismicity propagated from the borehole to this fracture and activated mainly the part of the fracture close to the borehole. We also observed the activation of an isolated cluster ca. 30 m away from the borehole. The second injection cycle seismically activated a sub-parallel fracture that seems connected to the former one, further away from the borehole. Details of the phase 1 stimulation are provided by Obermann et al. (2024) and Bröker et al. (2024a). After the phase 1 stimulations, we revisited selected intervals with larger injection volumes and revised injection protocols.

During phase 2 stimulation in interval 8 which was performed in June 2022 (selected experiment for this study), a total of about 274 m^3^ of water was injected over two injection cycles over two days with a total injection time of ca. 32 h with constant flow rate steps (Fig. 1a) (Bröker et al. 2023). An overnight shut-in was performed between the two injection cycles. A bleed-off step was skipped in order to maintain the stimulated volume pressurized. The flow rate-controlled protocol was utilized with ramp-up phases in which the flow rate gradually increased by increments of ca. 20 L/min, followed by a constant injection at the maximum flow rate. The maximum flow rate was 198 L/min in cycle 2, while the interval pressure reached a maximum of 17.8 MPa. During both cycles, the flow rate decreased abruptly a few times (specifically after ca. 3.5 and 11 h of injection in cycle 1, and after ca. 6, 8.5, and 13 h of injection in cycle 2), which were due to the clogging and subsequent filter replacement for one of the injection pumps. The operational details of the experiment can be found in Bröker et al. (2023). During this stimulation, 9368 events with high-quality picks were located (Obermann et al. 2024). The spatial extent of the seismic cloud exceeded 130 m. Several fractures were activated, covering different depth levels. From the first- to the second-injection cycle, the seismicity cloud generally shifted upward (Fig. 1d). The largest event in this experiment reached a magnitude of *M*_w_−1.64 (Obermann et al. 2024).

### Seismic Data Collection and Processing

To monitor the seismic activity generated by the stimulations, an ultra-high frequency network of 34 AE sensors and ACC was installed, covering a frequency band from 50 Hz to 100 kHz (Obermann et al. 2024; Bröker et al. 2024a; Plenkers et al. 2023). The open-source python-based DUGSeis package (Rosskopf et al. 2024c) was used to detect event candidates with a standard STA/LTA method based on the data from the five closest stations to the stimulated interval. On the snippet of the recorded waveforms by all sensors from each event candidate, a multi-frequency band higher-order statistics (kurtosis) picker developed by Poiata et al. (2016) was applied to pick P-wave onsets. In the second step, each pick was refined using an autoregressive-Akaike information criteria picker (Bagagli 2021; Leonard and Kennett 1999; Maeda 1985). The events with at least 5 picks were then located using a homogeneous medium with *Vp* = 5.1 km/s. Finally, a double difference event relocation method (Waldhauser and Ellsworth 2000) was applied to refine the hypocenter locations. The seismic catalog is available in Rosskopf et al. (2024a), and the procedure is described in Obermann et al. (2024) and detailed in Rosskopf et al. (2024b).

### Clustering and Fitting Planes to Seismic Events

To analyze the potential structures associated with seismic activity, we first group the seismic events into clusters based on their spatial proximity. Subsequently, we apply a plane fitting algorithm to each cluster to extract the potential planar faults or fractures represented by the seismic events. This two-step process allows us to map and characterize the geometrical orientation and slip tendencies of the activated planar structures based on the in-situ stress field. For clustering of the events, the event locations are subjected to standardization using the StandardScaler function from the Scikit-learn package (Pedregosa et al. 2011). Next, the custom function using the Density-Based Spatial Clustering of Applications with Noise (DBSCAN) algorithm (Cesca 2020; Ester et al. 1996) is implemented in Python using the Scikit-learn package (Pedregosa et al. 2011) to group the seismic events effectively based on the methodology proposed by Woodward et al. (2018). The Silhouette score is used as the metric to evaluate the quality of the clustering results. Utilizing the DBSCAN algorithm, each event is assigned either to a cluster label or recognized as noise (outlier). Based on the optimized outcomes derived from the Silhouette score, the initial normalized search distance (for evaluating neighboring points during the clustering process) was set at 0.3, with incremental increases of 10%. Additionally, the reduction factor for the minimum number of samples required to form a cluster was decreased by 25% over each additional iteration. The cluster numbers are then relabeled based on the distance of the center of each cluster from the middle of the injection interval. Next, the MSAC (M-Estimator SAmple Consensus) algorithm (Torr and Zisserman 2000), which is an extension of the RANSAC (Random Sample Consensus) algorithm (Fischler and Bolles 1981), is utilized to fit planes to seismic event locations in individual clusters. This process involves selecting points, estimating plane equations, and determining inliers. The algorithm iterates through clusters and updates fracture plane attributes until all feasible planes are identified. The points associated with each fitted plane are excluded from the next iterations, and each fracture plane is fitted independently of the previously fitted planes. Therefore, fitted planes can intersect each other. The threshold distances for fitting individual planes are set based on a sensitivity analysis for a range of distances and measuring the similarity between the fitted planes of each threshold in individual clusters based on the methodology proposed by Fadakar Alghalandis et al. (2013). The calculated threshold distance varies between 0.5 and 3 m for different clusters. In total, out of a total of 9368 events, 6143 events were fitted to 16 fracture/fault planes.

### Slip Tendency and Critical Overpressure Calculation

Based on stress measurements at various points within the reservoir volume (Bröker et al. 2024b; Ma et al. 2022; Bröker and Ma 2022), we have calculated stress gradients for the three principal stress directions. The extrapolated values for measured depths of 12 m and 400 m in borehole ST1 are *S*1 = 26.47, *S*2 = 25.43, *S*3 = 15.35 MPa, representing the vertical, maximum horizontal, and minimum horizontal stresses, and *S*1 = 33.76, *S*2 = 32.43, *S*3 = 19.58 MPa, respectively. Intermediate values at different elevations are linearly interpolated between these two data points. We assume that the stress directions are given by the far-field stress state estimated by Bröker et al. (2024b), where the direction of the minimum horizontal stress is orientated towards N22°E and the maximum principal stress is vertical. The inferred stress tensor is used to calculate the slip tendency over both pre-existing borehole-intersecting fractures and planes fitted to seismic events, defined as the ratio of the tangential stress to normal stress. It is then used to determine the critical pore pressure, defined as the excess pressure above static pressure required to initiate shear slip on corresponding fractures. We have utilized the methodology described by Mukuhira et al. (2017a, b), based on the Coulomb failure criterion for critical pore pressure estimation.

## Results

### THM and Seismicity

The thermo-hydro-mechanical (THM) and seismic data collected during the stimulation of interval 8 are presented in Fig. 1a–d. The pressure in the injection interval (measured downhole) reaches a maximum of 16.4 MPa during the first injection cycle and 17.8 MPa during the second injection cycle, representing an increase of 12.3 MPa and 13.7 MPa with respect to the initial static interval pressures, respectively (as shown in Fig. 1a). Approximately 4.6 h after the start of the first injection cycle (see Fig. 1b), interval 9 started to show a sharp pressure increase of 3.8 MPa. This shows the establishment of a stronger hydraulic connection between the injection interval and interval 9. Such a connection does not seem to be attributed to a packer bypass from within the borehole, since no temperature anomalies were detected in the data collected from the DTS cable installed along the ST1 borehole (as shown in Figs. 4a, 13b). In such a case, a direct fluid bypass would have been detected as a continuous flow of cold injection fluid entering the upper interval. Shortly after the pressure increase in interval 9, the pressure readings in ST1-interval 10, in MB8 at 123.9 m, and in MB2-interval 3 at ca. 186 m, show slight increases of less than 2 MPa shortly after hydraulic connection. Other pressure monitoring intervals in the reservoir volume (namely in MB2 (intervals 1–5), ST1 (intervals 10 and 11, i.e. the second and the third upper intervals to injection), and grouted pressure sensors in MB5 and in MB8) showed lower relative pressure increases (below a maximum increase of 5 MPa) as a result of injection in the stimulation interval. The outflow from the ST2 [the only cased, perforated (Meier et al. 2022), and open-to-atmospheric pressure borehole] was also monitored during the stimulation operation and showed a maximum increase of approximately threefold during the injection, from 15 L/min at ambient condition to ca. 38 L/min by the end of the injection in the second cycle. The response time of the ST2 outflow to variations in injection pressure decreased significantly, from nine minutes at the end of the first injection cycle to approximately one minute by the end of the second injection cycle.

Figure 1b which presents the seismicity rate, shows a sharp increase in seismic activity in the first injection cycle when the pressure increases by 8 MPa until the maximum of ca. 11 MPa is reached. A small drop in the pressure at 3.5 h after the beginning of the injection, triggers a significant decrease in the seismicity rate. Around 7 h after the beginning of the injection, we note an almost threefold decrease in the seismicity rate from its peak (coincided with the maximum recorded pressure increase in interval 9). During the shut-in period, the seismic activity is close to zero. During the second cycle, despite the higher injection pressure, the seismicity rate is lower compared to the first cycle. The start of significant seismic activity also appears at a higher pressure-increase of ca. 12.8 MPa.

The major anomalies—recorded by DTS and FBG sensors—in the reservoir volume are summarized in Fig. 1c. The results from MB7 are not presented in the figure, as MB7 did not show significant thermo-mechanical effects. The maximum FBG strain recorded in the reservoir volume occurred in MB8, during injection, at a depth of 173.7 m with extensional deformation of 76.9 micro-strain (the positive strain represents tensile deformation in this study). All FBG sensors in the reservoir volume, except five (with compressional deformation of larger than 5 micro-strain), mostly recorded extensional deformation during the injection experiment. Significant compressional deformations were observed in MB1 at 181.4 m and in MB5 at 194.3 m, with a max of -19.7 micro-strain and -13.3 micro-strain, respectively. The detailed temporal evolution of the recorded deformations and thermal anomalies by the DTS/FBG/DSS sensors are shown in Appendix 3. The temperature profile in the injection interval in ST1 shows the propagation of the cold fluid front towards the bottom of the interval during the first few hours of injection (Figs. 4a, 15). The propagation of the cold fluid’s front from the top to the bottom of the interval happens at a faster rate in the second injection cycle, suggesting enhanced reservoir transmissivity and potential reactivation of fractures in the deeper section of the injection interval (Bröker et al. 2023). The strain data from DSS, which covers the full boreholes’ length, captures significant compressional deformation at the deeper parts of the reservoir (Bröker et al. 2023) (Fig. 17), which is not observed in the FBG sensors. This could likely be due to either uncertainties in mapping of the DSS cable along the borehole, or the better spatial coverage of the DSS sensor. Notably, DSS in boreholes MB1 and MB8 showed maximum compressional strains of approximately − 1500 and − 1000 micro-strain, respectively, at measured depths of 244 m and 208.25 m (Fig. 17). These strains exhibited a continuous accumulation throughout injection cycles and with no major reversal pattern shortly after the injection stopped.

The establishment of the hydraulic connection between interval 8 (injection interval) and interval 9 is associated with several FBG sensors in MB8 showing a significant increase in extensional deformation (Fig. 16). Also, the depth ranges of 150–180 m in MB5 showed an abrupt change in the deformation pattern of FBG sensors, from compression to extension, mainly after the newly established hydraulic connection between intervals 8 and 9. In contrast, one of the FBG sensors in MB5 at 175.45 m, showed reversed deformation pattern about 10 min before the establishment of the hydraulic connection.

Figure 1c shows the positions of the identified major thermal anomalies within the reservoir volume during the stimulation operation (indicated by cross marks). The temperature anomalies reach approximately a maximum change of 1 °C in MB8. Overall, the variation pattern in temperature (whether it increases or decreases) can be strongly linked with the relative elevation of the observation point with respect to the elevation of the injection interval, depending on whether the flow direction is toward a shallower (colder) or deeper (warmer) section of the reservoir. Fluid movement from higher elevations (relatively colder zones attributed to the geothermal gradient) toward deeper sections of the reservoir generally resulted in a negative thermal anomaly at the observation point, and vice versa. The detailed time series of the observed thermal anomalies are illustrated in the following section.

Figure 1d shows that the majority of events occur above the injection interval, and also, the events propagate towards further distances in the second cycle.

### Pressure Dynamics during Seismic Activity

**Fig. 2:**
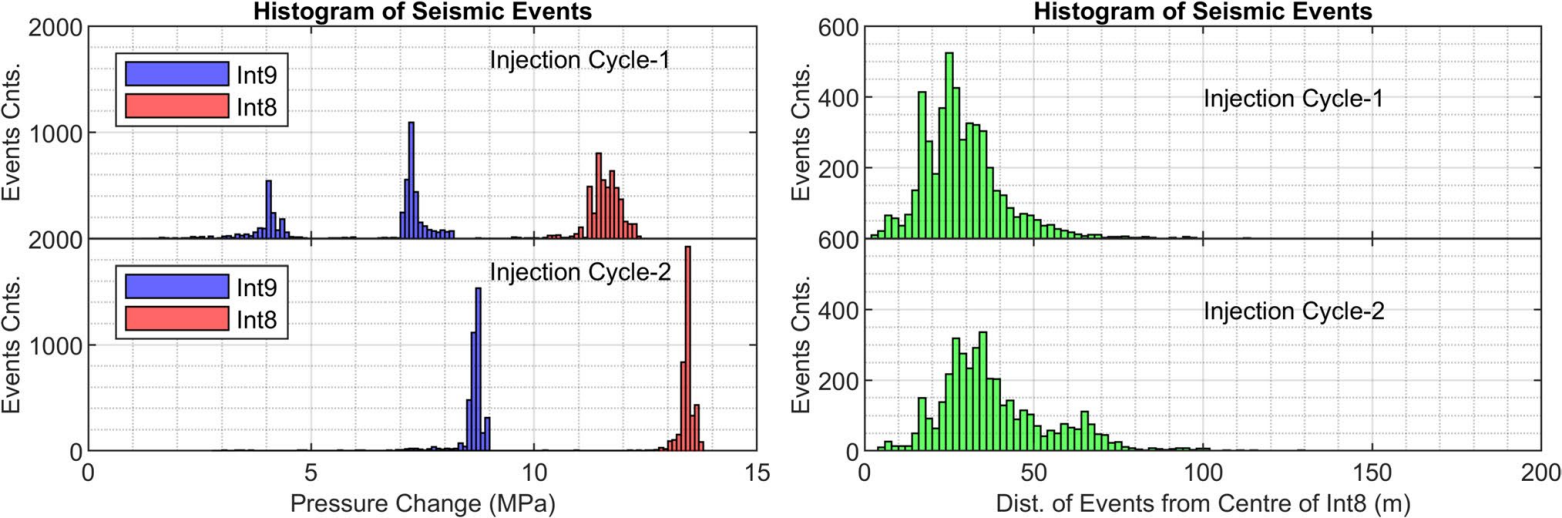
(Left): histogram of seismic events versus recorded pressure increase in the injection interval (8) as well as in the upper interval to injection (9) for each injection cycle, and (right): the histogram of the seismic events versus their Euclidean distance from the middle of the injection interval

Figure 2 presents the histograms comparing the counts of seismic events at different recorded pressure increases at the injection interval (8) and the upper interval (9) (shown on the left) and with respect to the Euclidean distance from the center of the injection interval (shown on the right). As shown in the pressure plot (Fig. 2-left), there is a significant shift in the distribution of injection over-pressure (in interval 8) and recorded pressure increase within interval 9 at the time of corresponding seismic activity, from the first injection cycle to the second. Notably, the recorded overpressures show a substantial increase in the second cycle within both intervals (8 and 9), and the distributions of the pressures shift from nearly a lognormal distribution in the first cycle to nearly a normal distribution in the second injection cycle. In the first cycle, a considerable number of events are also recorded at lower pressure in interval 9, compared with cycle 2. This is potentially linked to the initial pressure increase in interval 9 due to pressurization in the injection interval before a strong hydraulic connection between the two intervals is later established. The distribution of the Euclidean distance of the events from the center of the injection interval (Fig. 2-right) also shows changes in its statistics between the first and second injection cycles. Overall, during the second injection cycle, the events are slightly farther away from the injection interval. This can be attributed to an expansion of the stimulated volume. Furthermore, the first injection cycle displays a more unimodal distribution of events (centered at approximately 25 m distance), whereas the second injection cycle presents a more bi-modal distribution (centered at approximately 30 m and 65 m).

### Slip Tendencies of Activated Structures

**Fig. 3:**
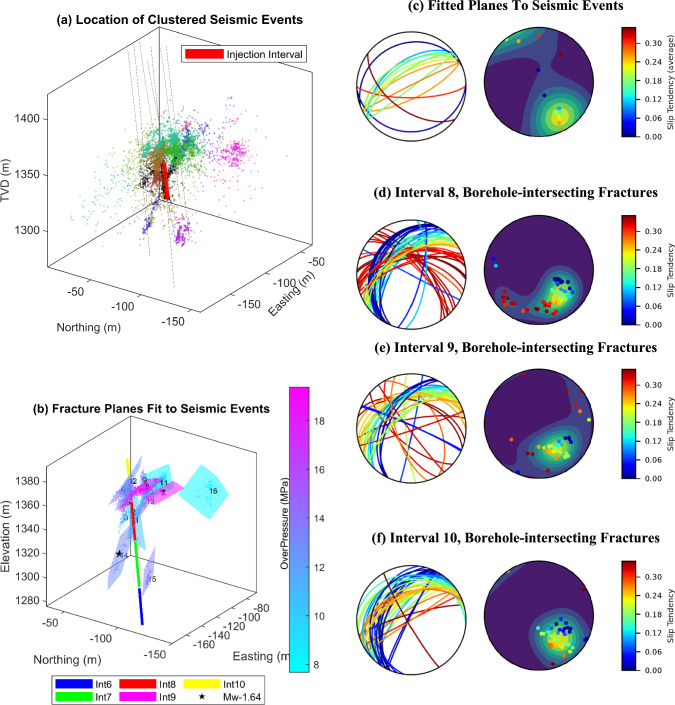
**a** clustered seismic events with all the drilled boreholes in Bedretto reservoir volume, **b** fitted planes to clustered seismic events colored based on the estimated average reactivation over-pressure for the corresponding planes calculated based on the slip tendency and in-situ stress state for each individual fitted structure, along with the intervals 6 to 10 in ST1, **c**–**f** the Stereonets (left) as well as the pole plots (right), colored with the estimated slip tendency of the corresponding structures for fitted planes, and natural structures in interval 8, 9, and 10, respectively (plotted with mplstereonet Package (Kington)). The color scale is consistent across all plots in c-f. (Colour figure online)

Figure 3 compares the slip tendency of the borehole-intersecting fractures/faults in intervals 8, 9, and 10 in ST1 with the slip tendency of the planes fitted to clustered seismic events. Figure 3a shows the 19 clusters of seismic events (excluding 152 non-clustered events). Figure 3b shows the fitted planes over the seismic event clusters colored according to the estimated average activation overpressure of each individual plane. The average activation overpressure was computed based on the estimated slip tendency over the corresponding plane and the in-situ stress state in the reservoir volume. A total of 16 planes are fitted to seismic events. The calculated overpressure represents the required pressure increase to trigger slip on the corresponding fitted planes. The majority of the seismically activated planes require less than about 14 MPa pressure increase for activation (relatively consistent with the maximum applied pressure increase in the injection borehole reaching ca. 13.7 MPa during the injections). In a few cases, however, the estimated activation overpressure exceeds 20 MPa, which appears to be unrealistic. This discrepancy can be attributed to either (a) an overestimation of the assumed friction coefficient, set at 0.6 in this study, (b) the possibility of a different mechanism contributing to seismic activity, such as mode-I tensile fracturing rather than mode-II shearing, or (c) uncertainties associated with stress estimation/perturbations in the reservoir volume.

Figure 3c–f compare the geometry and slip tendency of the borehole-intersecting fractures (in intervals 8, 9, and 10) as well as the fitted planes. In each of the three presented intervals, the borehole-intersecting fracture planes consistently display predominant patterns, striking mainly in the NE-SW direction as well as in the NW-SE direction (particularly in interval 8). The planes fitted to the clusters of seismic events also show a predominant NE-SW strike direction, although some planes are scattered in other directions. Although the predominant orientation of the fitted planes shows relatively high slip tendencies, the structures of other orientations exhibit higher values of slip tendencies.

### Thermal Anomalies

**Fig. 4:**
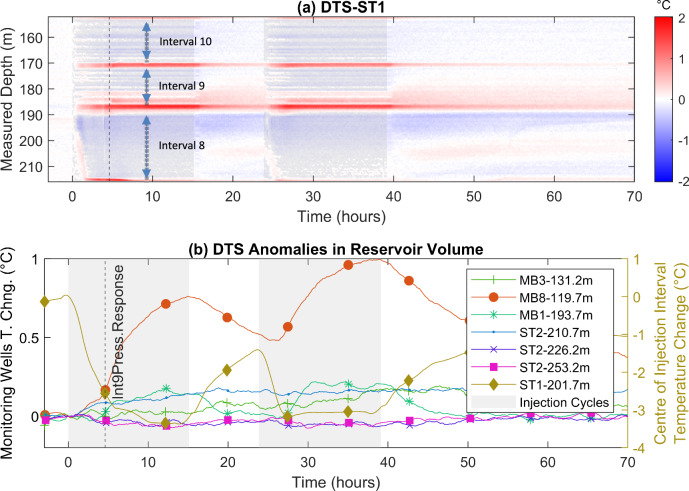
**a** The temperature change in time in ST1 intervals 10, 9, and 8 (de-trended along the measured depth for better representation, using a sliding window (Schweizer et al. 2021)), **b** the temperature-change time series at several monitoring locations with notable temperature anomalies recorded in the reservoir volume (denoised using the Savitzky-Golay filter (Savitzky and Golay 1964) in Matlab (The MathWorks Inc. 2023), and zeroed at the start of the injection in the first cycle. The two injection cycles are shaded with gray color in (**a**, **b**)

Figure 4a, b show the temporal evolution of the temperature profiles in the selected intervals of ST1 (8, 9, and 10) as well as at other borehole locations in the reservoir volume which show notable variations during the experiment, respectively.

Figure 4a shows the influence of the cold injection fluid (compared with the initial undisturbed condition) on the detected thermal anomalies. Also, the profile shows the sharp warmer fluid breakthrough at the lower part of interval 9 following the established hydraulic connection between the injection interval with interval 9. Several sharp and distinct temperature increases along ST1 during injection periods (at depths of ca. 170 m, 186 m, and 217 m) can be observed, which precisely coincide with packer locations. This can be attributed to two factors: (a) slight deformation of the packers due to dynamic pressure changes in the intervals that can generate frictional/compressional heating, which may not dissipate rapidly; and (b) increased pressure in the packers leading to compressing the DTS cable installed and protected within a sleeve in the packer, which may lead to apparent temperature increases caused by mechanical effects rather than actual heat. These temperature spikes dissipate rapidly after injection stops.

Additionally, a strong but brief positive temperature anomaly can be observed in the injection interval, with a distinct front just before the propagation of the cold injection fluid toward the bottom of the interval, which is likely caused by the flowing zone at ~ 204 m in ST1 (Interval 8) with positive natural thermal anomaly. This phenomenon is more evident at the same depth during shut-in periods, as this region warms up continuously, likely due to the inflow of naturally warmer fluid.

Figure 4b shows that the sampled and plotted fluid temperature at the center of the injection interval reaches a maximum drop of 3.3 °C compared with the initial static water temperature at the same depth, which is mainly attributed to the injection fluid’s downward movement from the wellhead during the injection. The observed temperature-drop recovers by a maximum of about 50% during the first shut-in period. Approximately 4 h after the start of the first injection, thermal anomalies were detected in the monitoring boreholes MB3, MB8, MB1, and ST2, with temperature increases of ca. 0.2 °C, 1 °C, 0.2 °C, and 0.2 °C at the measured depths of 131.2 m, 119.7 m, 193.7 m, and 210.7 m, respectively. The DTS system recorded only two minor cooling effects in the ST2 borehole, with slight temperature decreases of 0.05 °C and 0.08 °C at the measured depths of 226.2 m and 253.2 m, respectively, which are relatively close to the temperature interrogator’s precision (Silixa XT (Silixa XT)). The substantial reduction in the response time of the ST2 outflow to variations in injection pressure (as mentioned in the previous section), agrees well with the observed thermal anomalies and shows enhanced hydraulic connectivity between the two boreholes (ST1 and ST2).

Amongst all observed anomalies, the temperature profile in MB8 at 119.7 m has the most prominent increase, which peaks at the end of the second injection cycle. The rate of increase significantly accelerates after the established hydraulic connection between intervals 8 and 9 (the time of which is shown with a dashed line in Fig. 4b). The temperatures profile in other monitoring intervals are not significantly influenced by the established hydraulic connection.

### Hydraulic Connection to Intervals 9 and 10

**Fig. 5:**
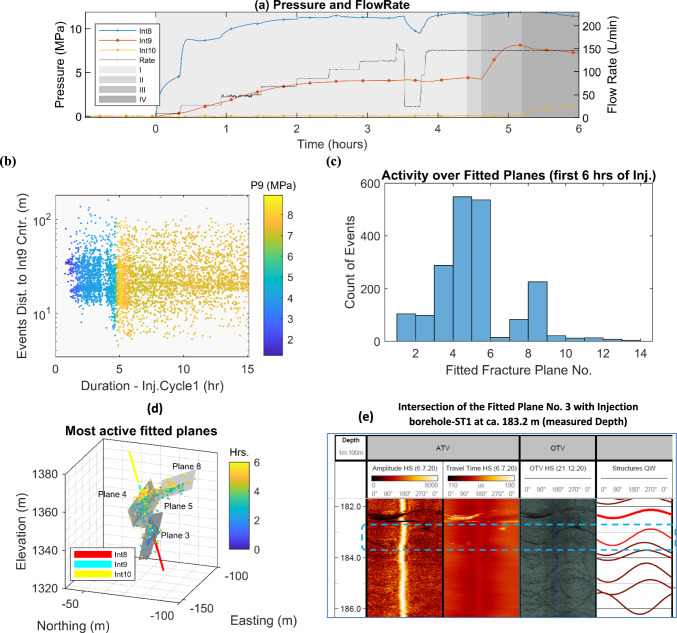
**a** Pressure change in intervals 8 (injection), 9, and 10, along with the injection flow rate, **b** spatial evolution of seismic events along with pressure with respect to interval 9, **c** histogram representing seismic activity over individual fitted planes to seismic events, **d** three-dimensional representations of the most active fitted planes along with the associated seismic events, and **e** the borehole-intersecting fracture within the injection borehole (interval 9), which aligns with fitted plane no. 3. **a**–**d** Present the data associated with the initial 6 h of the first injection cycle

Figure 5a–e shows the details of the establishment of the hydraulic connection between the injection interval (8) and the upper intervals during the first injection cycle. Figure 5a shows the four distinct phases of pressure evolution, marked with I, II, III, and IV, in the first 6 h of injection. As shown in the figure, during the first 4 h of injection in stage I, the pressure in the injection interval increases monotonically, however, relatively brief periods of decrease are also observed. After 4.4 h, in stage II, the injection pressure starts to decline, followed by a significant pressure increase in Interval 9 (at 4.6 h). As mentioned above, the absence of any DTS-detected cooling thermal anomalies in interval 9 (as shown in Figs. 4a, 15), rules out the possibility of a packer bypass. The pressure increase of interval 9 continues until ca. 5.2 h (the end of stage III in Fig. 5a), coinciding with intense seismic activity, leading to further strengthening of the hydraulic connection between the two intervals. Then, the pressure in interval 9 starts to decline significantly (stage IV). Subsequently, interval 10 shows a significant pressure increase. Figure 5b presents the Euclidean distance between the seismic events and the center of interval 9, along with the measured pressure in interval 9 corresponding to the time at which the seismic event occurred. The figure shows that the seismic events rapidly advance towards interval 9 followed by a significant pressure spike. Figure 5c shows a histogram of the seismic events over the active fitted planes within the first 6 h of injection. Amongst the total of 16 fitted planes, the figure shows that planes no. 3, 4, 5, and 8 (the geometry of which are shown in Fig. 5d) are the most active ones during this period. Plane no. 3 coincides with a borehole-intersecting fracture (see Fig. 5e), as inferred from borehole logs, at a measured depth of 183.2 m (at the lower part of interval 9). Despite the uncertainties associated with the logging depth, especially given that there exists another open fracture in the close vicinity of the one at 183.2 m, thermal anomalies based on DTS data (see Fig. 4a) further support that plane no. 3 is the main stimulated flow pathway related to the pressurization of interval 9.

## Discussions

In this section, we delve into the complex interactions observed between hydraulic connectivity, pressure variations as well as stress regime, and seismic event propagation. We will discuss how various properties influence the outcomes of stimulation, as detailed in the subsequent sections.

### Seismicity Pattern Versus Hydrostatic Pressure Regime

**Fig. 6:**
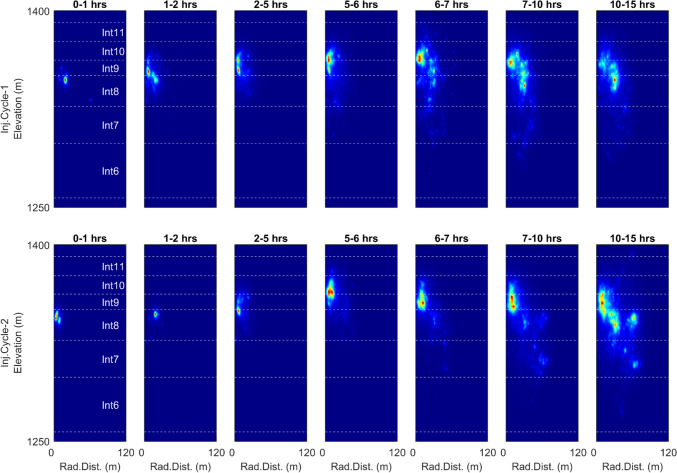
Temporal evolution of the spatial density of the seismic events in TVD directions versus the radial horizontal distance of the events from the middle of the injection interval, along with the boundaries of selected packed intervals. The density maps (normalized in each plot from 0 to 1) are generated using the Matlab density plot package (Malcolm 2024; The MathWorks Inc. 2023) and are shown for different time slots in each column during the stimulation injection for injection cycle 1 (upper row), and injection cycle 2 (bottom row)

To better visualize the relationship between the propagation pattern of seismic events and the pressurization of the reservoir volume, we have plotted the normalized spatial density of seismic events in the reservoir. For this purpose, the event's vertical elevation versus its radial distance from the middle of the injection interval (projected on the horizontal plane) for different arbitrarily selected time slots are chosen. The "data density plot" package in Matlab (Malcolm 2024; The MathWorks Inc. 2023) is used for this purpose. The results are shown in Fig. 6. The event densities can be compared with the pressures observed within intervals 8 and 9 (Fig. 1a). The boundaries of different packed intervals are also shown in Fig. 6 with horizontal dashed lines. It can be seen from the figure that the initiation of seismic activity begins around interval 8 at a small distance from the borehole (< 15 m) and then rapidly moves upward towards interval 9 (advancing toward the ST1 borehole) before propagating downward again at the later stage. This pattern is also repeated in the second injection cycle.

Similar patterns of seismic activity approaching backward toward the injection borehole from a distant location were observed in the Cotton Valley, Lower Frio, and EGS Collab projects (Schoenball et al. 2020; Phillips et al. 2002; Withers and Rieven 1996). Phillips et al. (2002) suggested that once slip initiates at a distant point, combined effects of local stress changes along the corresponding fracture as well as higher pore pressure at the wellbore location potentially cause reverse migration of events toward the injection borehole.

Another study by Yoshida et al. (2021), analyzing the M9 Tohoku-Oki earthquake (2011), suggested that the propagation of the seismic activity in the opposite direction to pressure can be linked to rate-and-state dependent friction, fault creep, and the associated stress redistribution. To the best of the authors' knowledge, there is limited discussion in the literature regarding such a reverse migration pattern of seismicity.

**Fig. 7:**
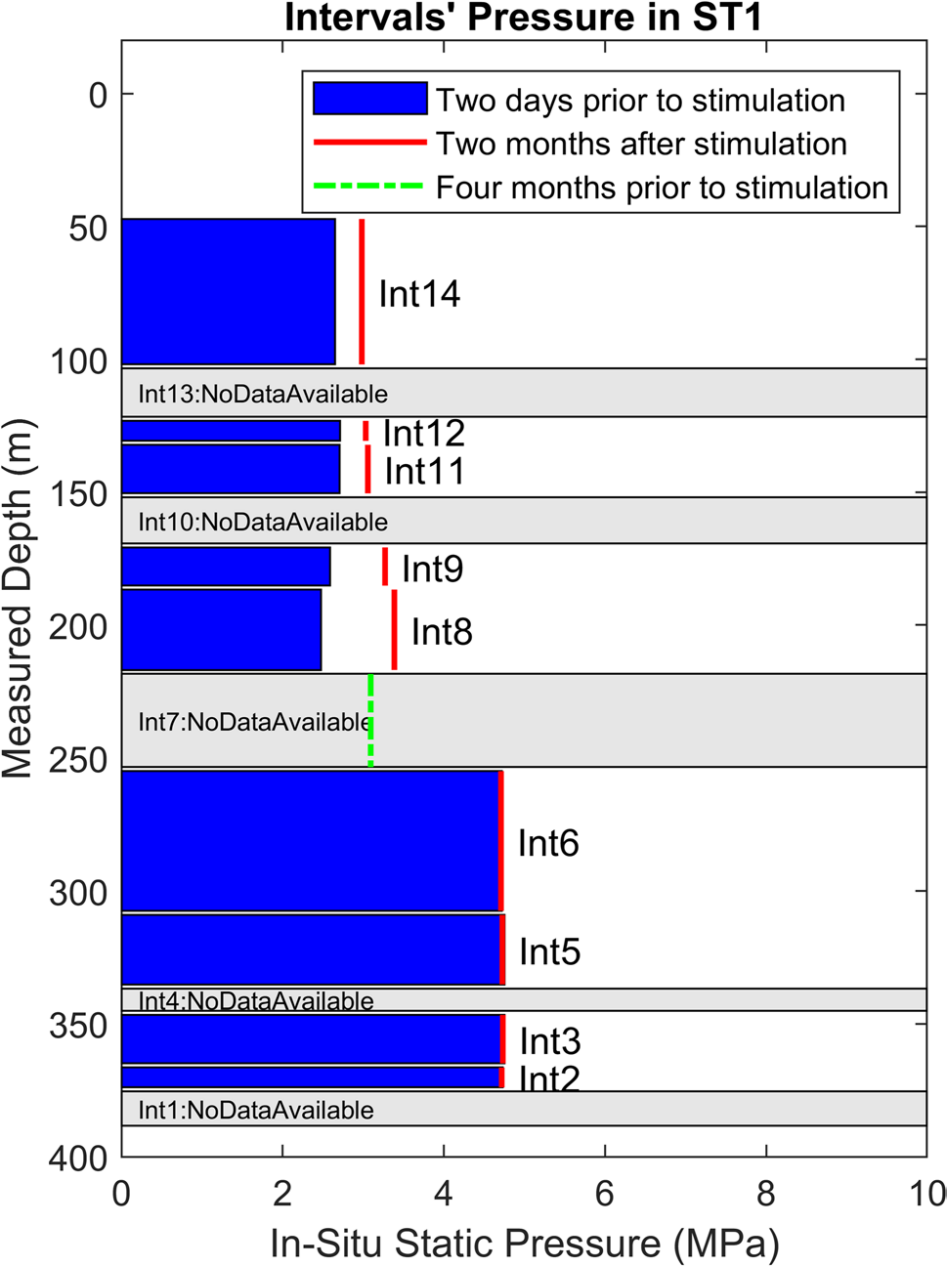
Static formation pressure (estimated at wellhead based on downhole pressure measurements and the hydrostatic columns of fluid at the center of the corresponding intervals) in individual packed intervals of ST1, recorded on June 20, 2022 (blue bar), and August 24, 2022 (red line), two days before and two months after the stimulation experiment, respectively. The last recorded pressure level before malfunctioning of the sensor in interval 7–i.e. four months before the experiment–is shown with green dashed line. The widths of the individual bars are equal to the interval lengths. Intervals without a working pressure sensor are shaded in gray color. (Colour figure online)

In this study, at the initial stages of injection, interval 9 is not significantly pressurized, due to the lack of a strong hydraulic flow path. This indicates that pressurization cannot be the cause of seismicity migration towards the injection borehole in this particular case. The shallower intervals in the injection borehole generally show lower hydrostatic pressure. This is illustrated in Fig. 7, which shows the static (wellhead) pressure in all intervals of the ST1 borehole, two days before the experiment, highlighting a substantial increase within interval 6 compared to the upper intervals (a difference of ca. 2 MPa). It is important to note that the pressure sensor in interval 7 started malfunctioning shortly before the experiment. However, readings from four months earlier showed an intermediate static pressure level in interval 7 between that of intervals 6 and 8. This also indicates that a strong sealing layer exists between interval 6 and the upper intervals. We argue that such pressure compartmentalization in the reservoir volume influences the pattern of pressure (and seismicity) propagation during injection, leading to an upward movement first (away from the injection interval). This is evident from the seismicity density plot (Fig. 6) as well as from pressure monitoring results during injection (Fig. 1a).

Pressurizing the upper interval creates a more balanced pressure distribution along the depth, especially after a strong hydraulic connection is established between intervals 8 and 9. Such a strong connection has the opposite impact on the seismicity pattern and will lead the seismicity downward and further away from the injection borehole (see Fig. 6). Specifically, after the hydraulic connection forms, the seismicity rate on the planes in which events advance towards the injection borehole significantly reduces and almost drops to zero, as will be further detailed in the following sections.

**Fig. 8:**
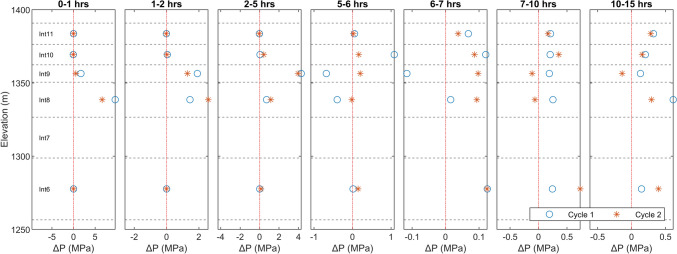
Temporal evolution of the recorded pressure change in packed intervals above and below the injection at different time intervals for the two injection cycles. Also shown in each plot are the boundaries of individual intervals at corresponding elevations. No data is shown for interval 7 due to a malfunctioning pressure sensor

To provide a more detailed quantification of the temporal and spatial pressure variations in the intervals above and below the injection interval, the pressure changes during the selected time intervals (as selected in Fig. 6) are plotted in Fig. 8 for both injection cycles. The results agree well with the observed seismicity migration patterns, indicating that pressure changes are more pronounced in the intervals above the injection interval during the initial hours of both injection cycles. In contrast, at later stages of injection, pressure increases are more notable in the lower interval, i.e. in interval 6.

Figure 7 also presents the static pore pressure distribution in ST1 (also measured at the wellhead) two months after the stimulation of interval 8. During this two-month pressure recovery period, a separate stimulation injection was conducted in interval 12, further above the injection interval (8). However, based on the recorded pressure profile, the stimulation of interval 12 only slightly affected the static pressures in intervals 11 and 14, with no considerable impact on interval 9 or below.

Figure 7 indicates a general increase in the hydrostatic pressure of all intervals above interval 6, particularly intervals 8 and 9. This is most likely caused by permanent rupture/leakage of the sealing layer (mentioned above) between intervals 6 and 8. This observation is corroborated by the recorded pressure data during the stimulation of interval 8 (Fig. 1a). Specifically, interval 6 did not show any noticeable pressure changes until approximately 6 h into the first injection cycle, after which it exhibited a relatively slight increase in pressure (maximum of ca. 0.5 MPa). In the second injection cycle, however, the pressurization of interval 6 occurred much earlier and with a larger amplitude, reaching a maximum increase of about 1.5 MPa. This suggests that a hydraulic connection, although relatively weak, was established between the injection interval and interval 6 following the compromised integrity of the seal between these two compartments.

According to the compartmentalized reservoir model presented by Byerlee (1993), when the seal between two compartments with significantly different pressure levels is ruptured, fluid movement from the high-pressure- to the low-pressure-compartment reduces the average effective normal stress across the compartments, potentially below the initial levels. Such pressure redistribution can trigger a sequence of earthquakes (Yamashita 1998) and lead to the breakage of the sealing integrity (Chang 2017).

Additionally, the largest recorded event during the entire experiment occurred approximately 1 h after the start of the pressure increase in interval 6 (during the first injection cycle). More importantly, this largest event is located on fitted plane 14 (Fig. 3b), the extrapolation of which intersects ST1 at the upper section of interval 7 and the corresponding borehole-intersecting fracture is identifiable from televiewer data. This observation can be well explained by the characteristics of the compartmentalized reservoir model proposed by Byerlee (1993).

In this study, although we did not directly observe the pressure response following the rupture of the sealing layer, due to the malfunctioning downhole sensor in interval 7, other evidences and observations (as mentioned above) agree well with the inferred rupture of the sealing layer above interval 6.

Although comprehensive geological modeling is needed to determine the exact geometry of the sealing layer and the impact of its rupture on the recorded event with the largest magnitude—an effort beyond the scope of this study—our observations show the importance of pressure compartments and their management through zonal isolation in such experiments. These factors play an important role in evolving the fluid flow and seismicity pattern during stimulation operations, particularly in EGS. For example, without the implemented zonal isolation, the pressure across all intervals of ST1, particularly between 6 and 9, would follow a hydrostatic gradient, potentially resulting in a significantly different seismic event propagation pattern and less intense seismic activity near interval 9.

This finding also has important implications for other applications such as cap rock integrity assessment during CO_2_ injections as well as in stimulation operations in oil and gas reservoirs. Optimal zonal isolation, however, can be challenging, particularly in geothermal systems because of the elevated temperature conditions (Norbeck et al. 2023), but several ongoing research projects are developing innovative solutions for effective and durable isolation in such environments (Hamm et al. 2021).

### Number of Injection Cycles versus Proximity of Activated Structures to Injection Interval

Another critical factor to consider when engineering the stimulation pattern of the reservoir is the number of injection cycles. As shown in Fig. 2, the statistical characteristics of the induced events, in terms of injection pressure and their distance, change significantly between the two injection cycles. During the second injection cycle, although there was no significant increase in the average distance of events from the injection well, the majority of events were triggered when the concurrently recorded pressures exceeded the maximum levels observed in the first cycle. This is a strong indication of the presence of a stress “memory effect” (Lavrov 2003), also known as the Kaiser effect (Kaiser 1953), which has also been observed in other experiments (Niemz et al. 2024; Kinscher et al. 2023; Kluge et al. 2021; Bohnhoff et al. 2010; Baisch et al. 2009).

**Fig. 9:**
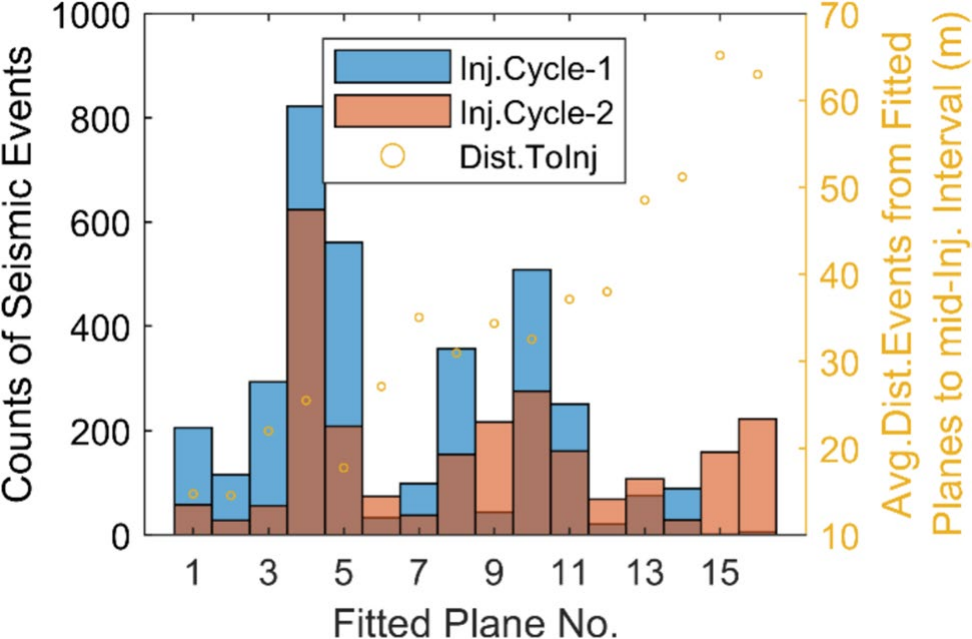
The histogram of the seismic activity on individual fitted planes during each injection cycle along with the average distance of the corresponding fitted planes to the middle of the injection interval

Figure 9 shows the distribution of the seismicity on all fitted planes along with the distance from the middle of the injection interval to the center of seismic events of the corresponding plane. The plane numbers are sorted according to their centers’ distance to the middle of the injection interval. It can be seen from the figure that all fitted planes show some level of seismic activity during both injection cycles, whereas generally planes closer to the injection interval are more active during the first injection cycle (in terms of the total number of events), and the planes further away from the injection (approximately beyond the threshold distance of ca. 40 m) are more active during the second injection cycle. This observation, combined with the significantly different events' distribution for recorded pressure magnitudes in intervals 8 and 9 during the second injection cycle (Fig. 2), can be attributed to the activation of larger parts of the same structures over the second injection cycle (that had yet to be activated in the first cycle). Such an activation pattern, which can also be a sign of the Kaiser effect, shows that regions with no prior seismic activity are more likely to be activated in response to pressure increase in the following injection cycles. This analysis implies that adjustments in injection pressure are necessary for subsequent injection cycles to effectively manage the stimulation pattern. However, the slight increase in the average distance of seismic events from the injection interval between the two cycles, despite the use of higher pressures in the second cycle, shows that the extent of the stimulated reservoir volume is influenced more by the existing fracture geometries and rock mechanical properties rather than by pressure (given the relatively similar duration of stimulation injections in both cycles). This shows the importance of performing detailed geological and geophysical surveys, combined with real-time analysis of reservoir data during the first injection cycle, to optimally design subsequent stimulation cycles.

**Fig. 10:**
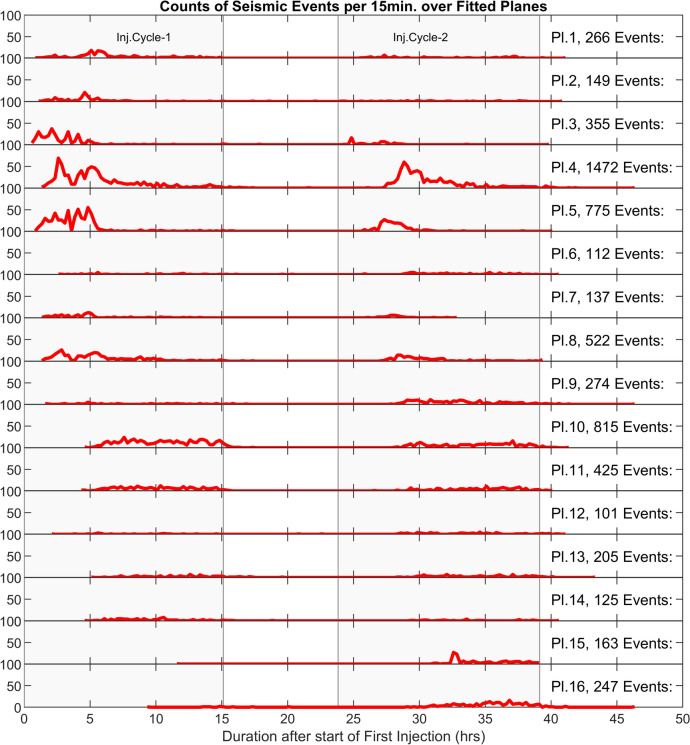
Time series representing the rate of seismic activity over individual activated structures in counts of events per 15 min during the whole stimulation period (including the shut-in phases). The total number of recorded events on each fitted plane is shown in individual plots. The two injection cycles are shaded

To compare the details of seismic activity over all fitted planes, the corresponding temporal evolution of seismic activity rates over individually fitted planes is shown in Fig. 10. This analysis covers the whole experiment including both injections and shut-in cycles. In both injection cycles, the rate of seismic activity across all fitted planes shows local increases and decreases, likely attributed to the stepwise propagation of the stimulation front. Additionally, planes closer to the injection interval show a peak in seismic activity over both injection cycles, whereas planes further away display a relatively more uniform rate of activity without a clear peak. For example, plane 10, shows relatively uniform seismicity during the first injection cycle followed by a period of relatively strong seismic activity immediately after the first shut-in period. This can be potentially attributed to either: (a) a more significant Kaiser effect in regions closer to the injection interval, (b) the lower rate of pressure change and, therefore, seismicity further away from the injection interval, or (c) reverse pressure fluctuation effect also known as Noordbergum effect (Verruijt 1969) [which can be caused by either more rapid propagation of mechanical effects compared with hydraulic effects, or amplified mechanical deformation in below a "relatively soft aquitard" (Kim and Parizek 1997)] that can briefly increase the pressure following the shut-in and therefore might potentially induce more seismicity. Finding the exact cause of such a seismicity pattern, in particular, on plane 10, needs a comprehensive analysis beyond the scope of the current investigation.

Given that plane numbers increase with their distance to the injection interval, the general activity pattern shows that further away planes are activated at the later stages of stimulation injection. Figure 10 also shows that, in both injection cycles, the seismic activity first starts in plane no. 3. The planes 3, 4, 5, and 8 show the sequencing of the start of seismic activity in the first injection cycle, progressing from planes 3 to 5 and 4, and then to 8. The seismic activity starts further away from the injection interval and then moves towards the upper intervals of the borehole (from deeper regions of the corresponding fitted planes to shallower parts). This shows the existence of a natural and already permeable flow path intersecting interval 8, which causes the fluid to initially travel further away from the borehole before reaching plane 3 and subsequently the other planes. This observation is consistent with the significantly high recorded transmissivity of interval 8 (2.8E−07 m^2^/s) before the start of the stimulation operation.

During the first injection cycle, generally the initiation of seismic activity can be divided into four groups of planes—i.e., planes 1–5, 6–9, 10–14, and 15–16—with each group exhibiting almost simultaneous initiation of seismic activity. Within each group, however, the exact temporal sequence of plane activation initiation did not necessarily follow the ordering of the planes' distance to the injection point. For example, planes 1 and 2 (closest to injection) were significantly activated after plane 3.

In contrast, the second injection cycle showed a significant change in activation pattern. Closer to the injection interval, the start of the seismic activity was not happening as a function of the distance of the plane to the injection interval, whereas further away planes (from plane 6 onwards) demonstrated a sequential start of seismic activity with respect to the distance of the corresponding planes to the injection interval. These observations suggest that during the first injection cycle, seismic activation in individual groups might have been mainly influenced by the formation of new hydraulic pathways that linked new reservoir sections to the injection interval. Whereas in the second cycle, the propagation of pressure through the pre-established hydraulic system (from the first cycle) is likely dominating the activation pattern.

It is also observed that seismic activity over plane 3 peaks and diminishes rapidly after the establishment of a new hydraulic connection between intervals 8 and 9 in the first cycle. Despite an increase in maximum injection pressure during the remaining part of the first injection cycle, seismic activity did not recover significantly on this plane. However, during the second injection cycle, plane 3 shows a relatively considerable level of seismic activity at a pressure level which is even below the maximum injection pressure recorded in the first cycle. This highlights a potential resetting of the Kaiser effect in the second injection cycle. In a recent study, Kim and Avouac (2023) used a statistical model to describe the seismicity rate based on the convolution of the injection history in Otaniemi EGS in Finland. They found that various injection locations at the different stimulation phases can result in stimulating new rock volumes and, therefore, leads to new hydraulic flow pathways as well as to changes in effective stress distributions, which can potentially mute the Kaiser effect. This phenomenon (newly generated flow pathway) is also the most likely cause of the observed seismic activity pattern in plane 3 during the second injection cycle. This represents the significant impact of the altered hydraulic system on the evolution of the seismicity and stimulation pattern. Additionally, these findings show the critical role of zonal isolation in managing the pressure compartments of the reservoir before injection operations, which is essential for the dynamics of reservoir stimulation.

### Prevailing Stress Regime

The slip tendency analysis, which was performed on natural fractures/faults intersecting the injection interval, and intervals 9 and 10, as well as over the stimulated (fitted) planes (Fig. 3), shows that, despite the presence of a pattern oriented in a northwest–southeast/east–west (NW–SE/E–W) direction with a substantially large slip tendency (maximum estimated range), the majority of the stimulated planes are oriented in a northeast-southwest (NE–SW) direction. This geometry also agrees well with the dominant borehole-intersecting fracture pattern observed from geophysical borehole logs. The consistent activation of planes with relatively similar geometry, in particular at longer distances from the injection interval (Fig. 3b), strongly indicates the existence of prevailing stress conditions within the reservoir volume, which agrees well with the results presented by Ellsworth et al. (2019).

In addition, it is observed that only a minimal number of natural structures intersecting the injection borehole, are activated during the stimulation injections. Consequently, very limited seismic activity was recorded at a close distance to the injection interval. Specifically, only 25 events were recorded within a 5-m distance from the middle of the injection interval throughout the entire stimulation experiment. A similar phenomenon was reported during a stimulation experiment in Cooper Basin, Australia, by Baisch et al. (2009), where the immediate vicinity of the injection borehole remained seismically quiet during restimulation. They attributed this to the Kaiser effect (Lavrov 2003; Kaiser 1953) following the preceding stimulation experiments in the same volume. However, in the present study, we already observed similar behavior (very limited seismic activity near the injection interval) even during the first phase of stimulation (phase 1), which rules out the Kaiser effect as the main cause for the observed phenomenon. On the other hand, previous studies have shown that the stress distribution, particularly near the drilled boreholes, can be significantly perturbed due to geomechanical effects. These effects can impact both, the magnitude and orientation of stresses up to a distance of approximately 10 to 15 times the borehole radius (Weijermars 2013). In our study, we argue that the low level of seismic activity in the near-borehole region is caused by stress perturbations around the borehole. Such findings underline the necessity for a comprehensive investigation of stress perturbations around drilled boreholes when designing an efficient stimulation protocol.

Figure 3b, shows that three fitted planes exhibit over-pressures (required for activation) that even exceed the injection levels. Several factors, including mixed-mode fracturing, uncertainty in a stress state, dynamic stress perturbations, geological conditions, and lower-than-expected friction coefficients may contribute to this difference. It is noticeable that these three planes are oriented more horizontally than the others. Since we interpreted a normal to strike-slip faulting stress regime in the reservoir volume that favors the reactivation of steeply dipping to vertical fracture planes, nearly horizontal planes have low slip tendencies and high critical over-pressures.

In such a stress regime, hydraulic fractures would initiate vertically rather than horizontally. It is unlikely that the overburden stress is the lowest principal stress in our reservoir volume, which is a prerequisite for horizontal hydraulic fractures. Therefore, it is unlikely that the horizontal fractures are activated under the mode I (tensile) regime.

Similar near-horizontal seismic activity, which was not fully explainable by the in-situ stress state, has been observed in other studies. Warpinski and Teufel (1987), Phillips et al. (2002), Roche and van der Baan (2015), and Kwok et al. (2020) demonstrated that features such as faults, joints, bedding planes, stress contrasts, stratigraphic changes, and sedimentary beddings can significantly influence fracturing behavior, potentially contribute to horizontal activated features, and therefore to fracture containment within specific layers. However, in our study, the geometry of the three observed near horizontal fractures varies significantly over a small scale (variation of more than 100° in dip angle over a distance of approximately 30 m), making it unlikely that specific structure layering effects are responsible for the observed phenomena.

Additionally, the observed near-horizontal fractures do not seem to act as boundaries within the seismicity cloud, as events and fitted planes exist both above and below them, which further suggests that layering effects are not the factor responsible for near horizontally activated structures. Instead, we argue that the observed exceedingly large overpressure over the three fitted planes can be attributed to the change in static stress (Hardebeck et al. 1998) caused by other planes’ activations. For example, plane 8, which has the largest estimated activation over-pressure amongst all 16 fitted planes, is likely intersecting plane 9. In addition, the large estimated overpressure on the few planes may also be attributed to the stress heterogeneity (especially at the same depth) and variation of the friction coefficient within the reservoir volume, which are not considered in this study to estimate critical overpressure based on the methodology proposed by Mukuhira et al. (2017a). Bröker et al. (2024b), based on the analysis of mini-frac tests conducted around the BedrettoLab, observed that stress heterogeneities can range from less than 10 m along individual boreholes to hundreds of meters between different boreholes. Therefore, local stress heterogeneities exist on multiple scales, potentially due to the presence of large fault zones within the rock volume (Zhang et al. 2023), as well as dynamic perturbations in the stress field, such as change in static stress from previous fracture reactivation. These heterogeneities may introduce uncertainties into our slip tendency analysis, particularly in the case of the three observed near-horizontal activated planes. However, despite the existing uncertainties in overpressure estimation, the results align generally well with experimental observations.

**Fig. 11:**
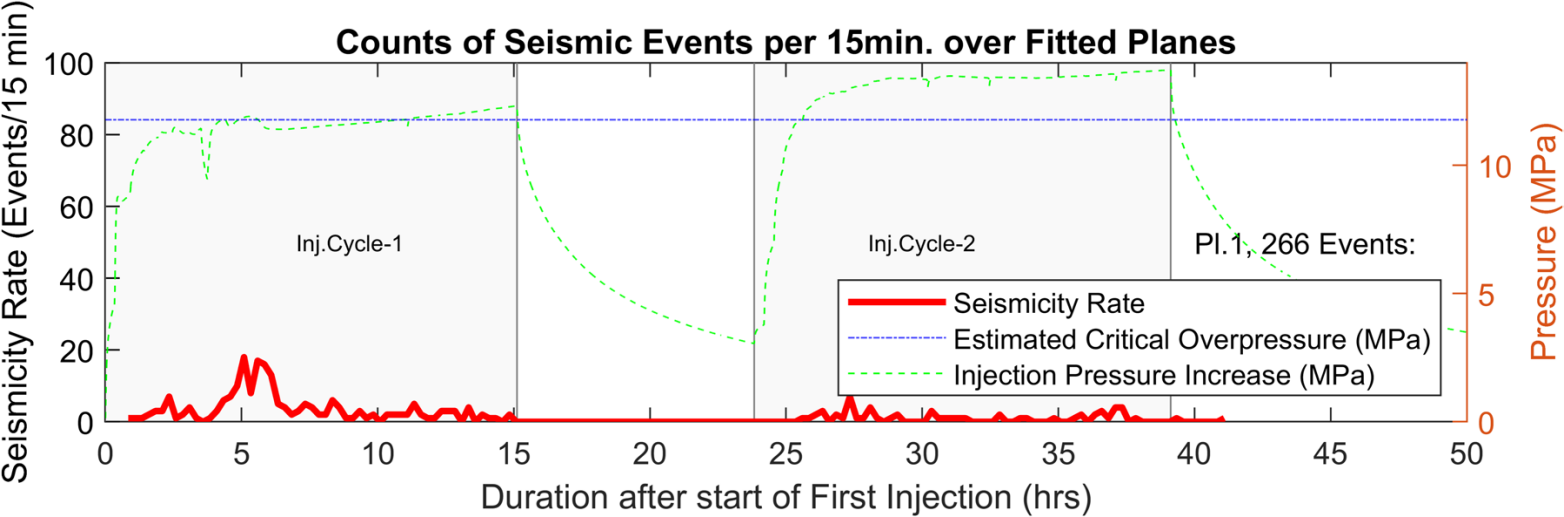
The seismicity rate as well as the estimated critical overpressure for fitted plane no. 1, alongside the pressure increase profile in the injection interval (8)

**Fig. 12:**
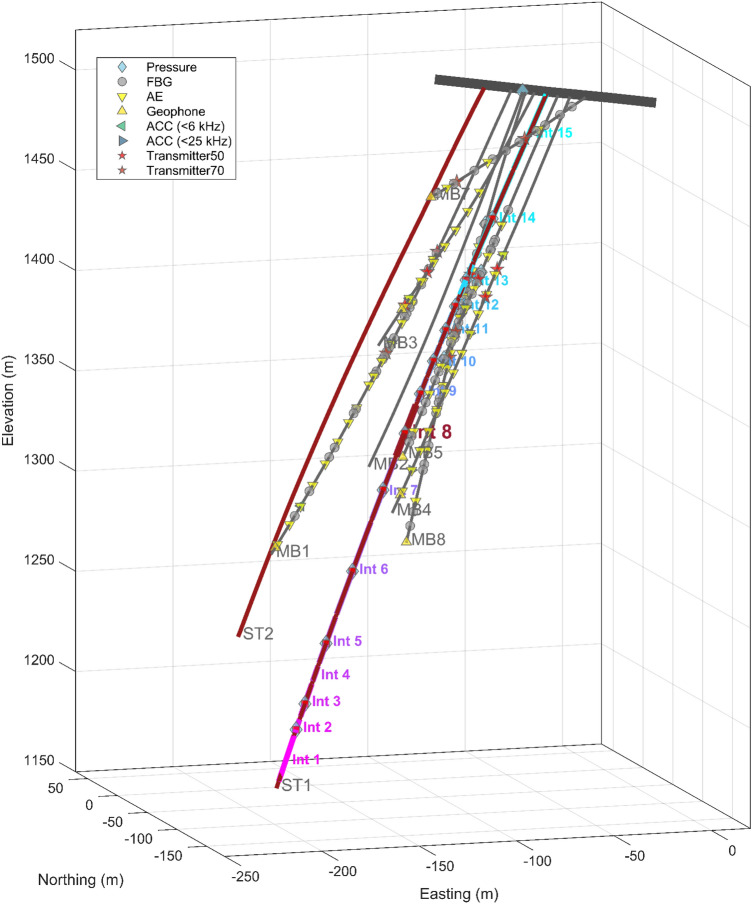
A schematic representation of the borehole geometries at BedrettoLab along with different sensors’ location/type. The packers and pressure monitoring intervals in MB2 are not shown

**Fig. 13:**
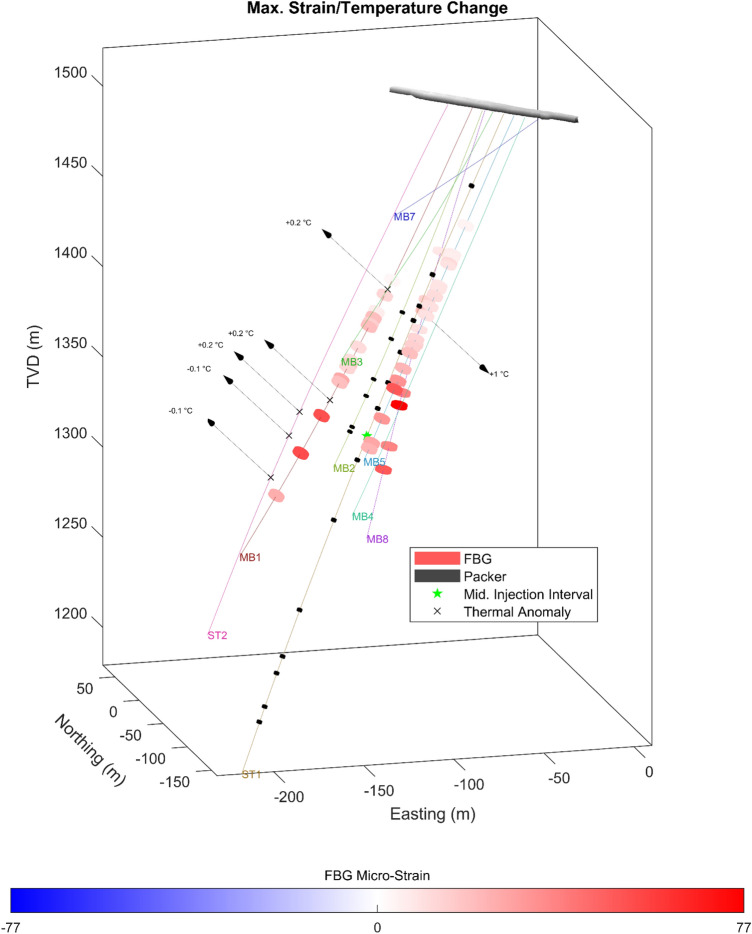
Enlarged view-1 of Fig. 1 in the main text, with a schematic representation of the BedrettoLab with the drilled boreholes, packer locations in ST1 and MB2, location of the detected thermal anomalies, along with the maximum FBG-strain/temperature changes recorded in the reservoir volume during the experiment (FBG markers are colored according to the strain scale shown in the colorbar, although the legend shows only a representative red color)

**Fig. 15:**
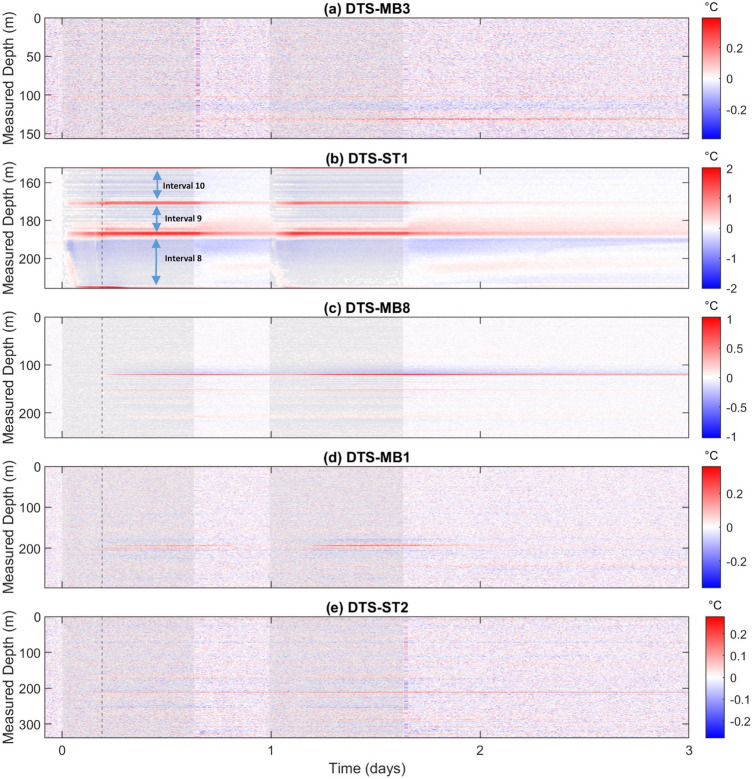
The temperature change in time profile (de-trended along the measured depth for better representation, using a sliding window (Schweizer et al. 2021)) in: **a** MB3, **b** ST1 (only over intervals 8, 9, and 10), **c** MB8, **d** MB1, and **e** ST2. The vertical dashed lines mark the initiation of pressure spike in interval 9 (upper interval to injection) after the establishment of a strong hydraulic connection with the injection interval. The temperature profiles in the boreholes without notable changes are not displayed. The zero time in *x*-axes of all sub-plots represent the beginning of the injection in the first cycle and the shaded areas show the two injection cycles

**Fig. 16:**
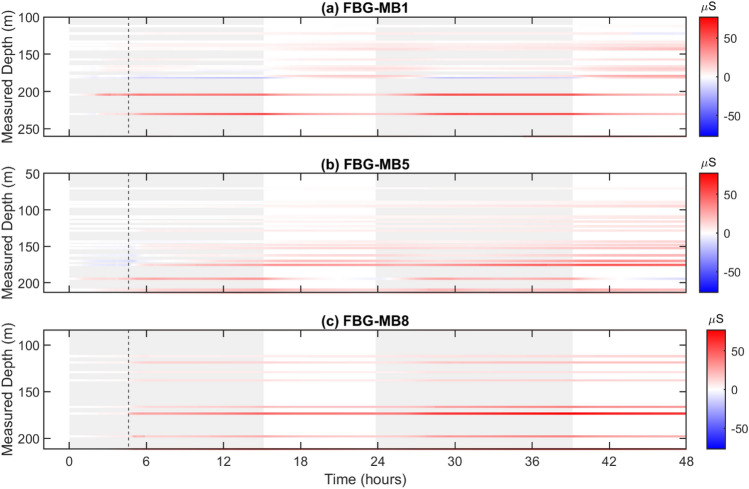
Recorded strains using FBG sensors from three wells in the reservoir volume with notable changes, namely: **a** MB1, **b** MB5, and **c** MB8. The negative strain represents compression. All strain values are zeroed at the beginning of the injection in the first cycle to better show the relative changes caused by stimulation injection. The vertical dashed lines mark the time at which the pressure of interval 9 (upper interval to injection) sharply increased after the establishment of the strong hydraulic connection to injection interval. The zero time in *x*-axes of all figures represent the beginning of the injection in the first cycle. The shaded areas mark the two injection cycles

**Fig. 17:**
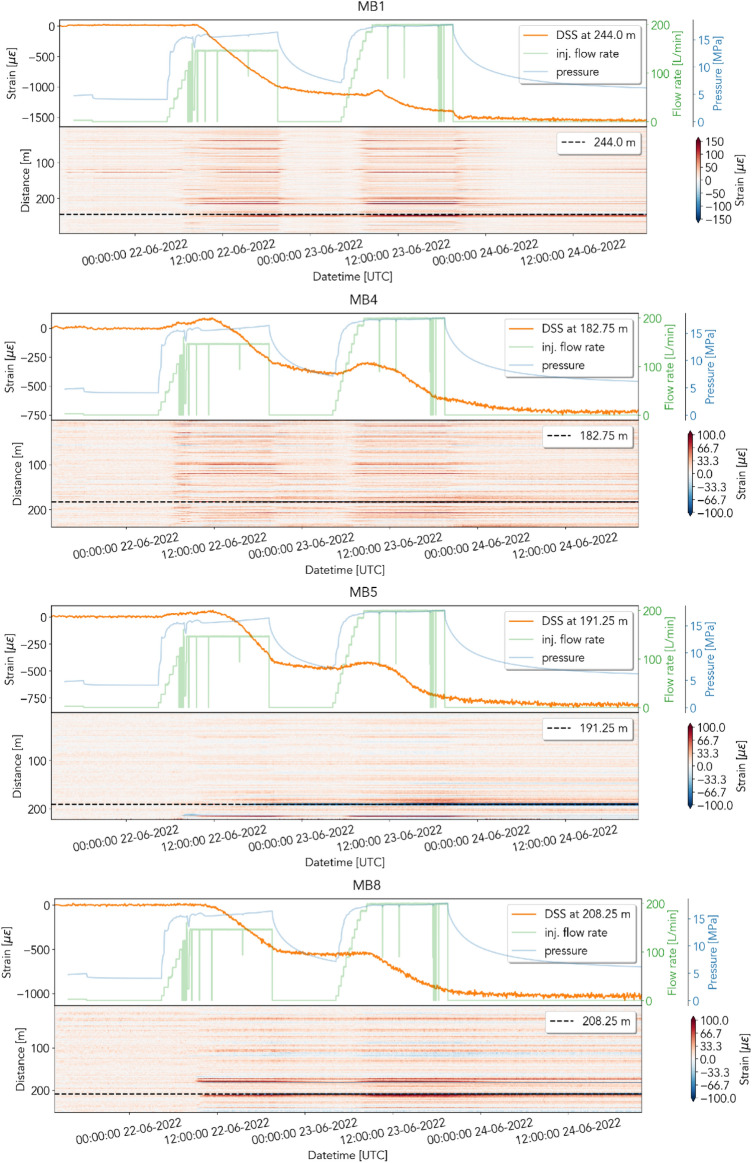
Recorded strain time-series from DSS sensors in all monitoring boreholes with major changes (after Bröker et al. 2023). Time is presented in UTC. The upper panel of each sub-figure displays selected strain time series at locations where maximum compression along the corresponding boreholes were observed, alongside measured injection pressure (blue) and injected flow rate (green) profiles for comparison. The lower part illustrates waterfall plots of DSS measurements along the full borehole length over time. DSS strain data are zeroed at the beginning of injection in the first cycle. For clarity, line plots represent selected peak strains at specific depths that exceed the scale limits of the waterfall plots (to have a better view of other significant but less extreme strain data in the waterfall plots). (Colour figure online)

While, ideally it would have been possible to validate overpressure estimates against pressure data from sensors in MB5, MB8, and MB2, the complex fracture network, its behavior under high-pressure injections (with potential elastic/permanent opening/shearing), and the relatively much lower seismic activity in the very close vicinity of the boreholes in our experiment, further complicate such comparisons. However, for plane 1, which is the closest to the injection interval (intersecting the injection interval) and also one of the main flowing zones in the interval, we plotted the injection pressure increase in the interval and the seismicity rate together with the estimated overpressure required for activation of this particular plane. The results are shown in Fig. 11. As shown in the figure, the peak in seismicity rate in the first injection cycle, which is also the maximum rate on this plane during the whole experiment, matches very well the timing at which the pressure-increase in the injection interval reaches the estimated critical overpressure for activation of this plane. Extending this analysis to other more distant planes is, however, challenging due to the lack of direct monitoring data near seismicity locations as well as dynamic hydromechanical changes in the reservoir volume during the progress of the stimulation operation. Also, the rupture of the seal between injection interval and interval 6 (as mentioned above), which occurred approximately six hours after the start of the injection in the first cycle, is likely to introduce significant hydromechanical changes in the reservoir volume that can change the spatial stress distribution significantly.

In general, our results show that planes striking NW–SE exhibit relatively lower estimated critical overpressures, consistent with observations of dominant activated structures from other experiments in the same reservoir volume (Obermann et al. 2024). Yet, some planes with relatively similar spatial extents show markedly different overpressure estimates. This suggests that changes in static stress, resulting from fracture opening and shearing, may significantly influence activation patterns, potentially more than stress field uncertainties within the reservoir volume. In the future, performing stress inversion using moment tensors as well as a detailed investigation of the uncertainties associated with friction coefficient could help resolve the stress heterogeneity and excessively large estimation of critical over pressure on some of the activated planes within the volume.

## Conclusion

This study investigated the reservoir’s THM response during stimulation operation to understand the relationship between geomechanics and stimulation patterns.

We see that the zonal isolation in the injection borehole plays a crucial role in influencing the propagation pattern of stimulated volumes, in particular leading to the alteration of hydraulic connectivity within the reservoir.

The spatial distribution of seismic activity varied significantly depending on the cycle and distance from injection. Seismic activity during the second injection cycle initiated at pressure change levels exceeding those that induced seismicity during the first cycle, a clear indication of the influence of the Kaiser effect within the reservoir. The study also demonstrates that the Kaiser effect may be modified by changes in the reservoir hydraulic system during stimulation as a result of flow path alterations/modifications.

Despite the presence of borehole-intersecting fractures with large slip tendencies, the stimulated planes exhibit different orientations with slightly lower slip tendencies. This may be partially attributed to stress perturbations at the proximity of the borehole.

Distributed temperature monitoring in the reservoir volume, in particular within the injection borehole, helped us to locate the main activated structure contributing to the hydraulic cross connection between different packed intervals of the injection borehole (i.e., intervals 8 and 9).

Comparison between injection cycles revealed significant changes in seismic event characteristics, showing that the extent of stimulated volume can be affected by adjusting the number of injection cycles as well as the maximum injection pressure. The findings emphasize the advantages of real-time evaluations during injections, showing their importance in designing and streamlining optimized stimulation operations. Also, implementing the stimulation process in multiple stages would be beneficial, to gain a comprehensive understanding of the reservoir's response during the initial cycle(s). This insight can then be used in the design of the injection protocol for subsequent cycles.

Further investigations of factors like mixed-mode fracturing, friction coefficients, and uncertainty in dynamic stress changes are recommended for a comprehensive understanding of the processes involved in reservoir stimulation experiments.

## Figures and Tables

**Fig. 14:**
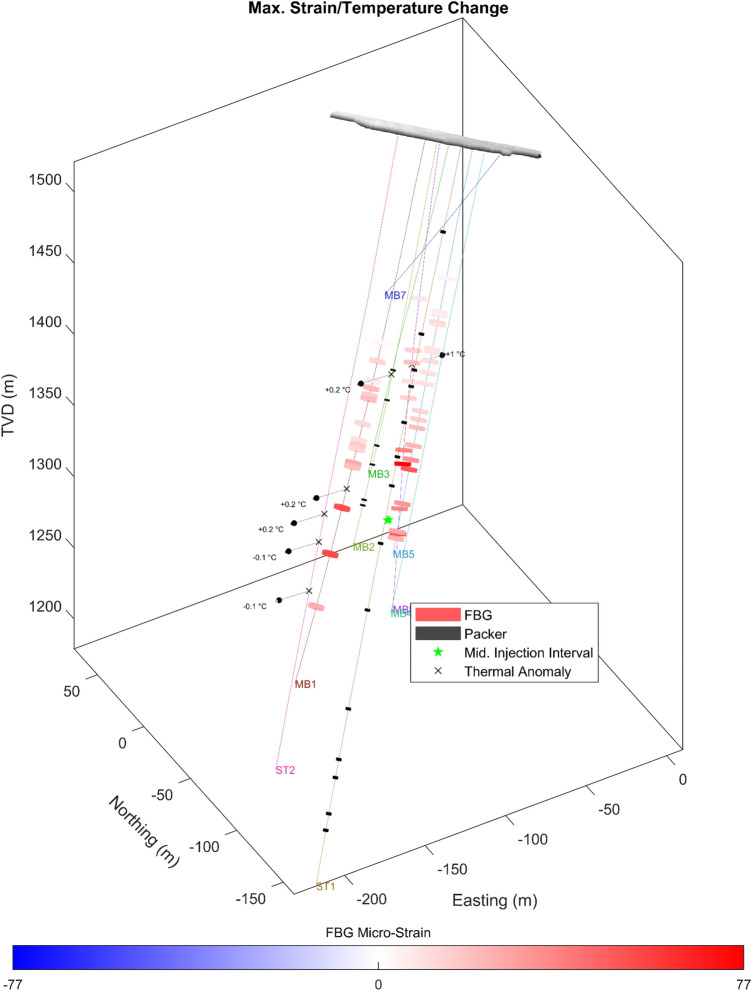
Enlarged view-2 of Fig. 1 in the main text, with a schematic representation of the BedrettoLab with the drilled boreholes, packer locations in ST1 and MB2, location of the detected thermal anomalies, along with the maximum FBG-strain/temperature changes recorded in the reservoir volume during the experiment (FBG markers are colored according to the strain scale shown in the colorbar, although the legend shows only a representative red color)

## Data Availability

The datasets used for the current study are available from the corresponding author on reasonable request.

## Appendix 1: Boreholes/Sensors Network in BedrettoLab

See Fig. 12.

## Appendix 2: Enlarged View of Fig. 1

See Figs. 13 and 14.

## Appendix 3: Detailed Recordings by Optical Fibers in the Reservoir Volume During Stimulation Experiment

See Figs. 15, 16 and 17.
